# Complexity thinking as a guide for good, evolving, and disruptive obstetric practices

**DOI:** 10.1590/0034-7167-2025-0504

**Published:** 2026-08-21

**Authors:** Roseleia Regina Halmenschlager, Marli Terezinha Stein Backes, Janet Mercedes Arévalo-Ipanaqué, Dirce Stein Backes

**Affiliations:** I Universidade Franciscana. Santa Maria, Rio Grande do Sul, Brazil; II Universidade Federal de Santa Catarina. Florianópolis, Santa Catarina, Brazil; III Universidad Peruana Cayetano Heredia. Lima, Peru

**Keywords:** Nonlinear Dynamics, Obstetric Nursing, Patient Care Team, Social Identification, Natural Childbirth., Dinámica no Linear, Enfermería Obstétrica, Personal de Atención al Paciente, Identidad Profesional, Parto Normal.

## Abstract

**Objectives:**

to reflect on good obstetric practices in light of complexity thinking.

**Methods:**

a theoretical-reflective essay that considered classic bibliographic sources and contemporary authors who defend the evolutionary dynamics of adaptive systems and, in parallel, provide theoretical-systemic support to the complex and inseparable phenomenon of “pregnancy, childbirth, and birth”.

**Results:**

by understanding pregnancy, childbirth, and birth as inseparable phenomena and integral parts of complex adaptive systems, it becomes possible to overcome disciplinary barriers, reduce hierarchical asymmetries, and promote horizontal, collegial, and mother-child dyad-centered care practices.

**Final Considerations:**

theoretical reflection on good obstetric practices, in light of complexity thinking, gives rise to disruptive, evolutionary, and sustainable formative paths, capable of overcoming disciplinary barriers and establishing horizontal, collegial, and systemic intervention processes in obstetrics.

## RESUMEN

**Objetivos:**

reflexionar sobre las buenas prácticas obstétricas a la luz del pensamiento complejo.

**Métodos:**

es un ensayo teórico-reflexivo que consideró fuentes bibliográficas clásicas y contemporáneas que defienden la dinámica evolutiva de los sistemas adaptativos y, al mismo tiempo, brindan un apoyo teórico-sistémico al fenómeno complejo e indisociable de “gestación, parto y nacimiento”.

**Resultados:**

al comprender el embarazo, el parto y el nacimiento como fenómenos inseparables e integrantes de sistemas adaptativos complejos, es posible superar las barreras disciplinarias, reducir las asimetrías jerárquicas y promover prácticas asistenciales horizontales, colegiadas y centradas en la díada madre-hijo.

**Consideraciones Finales:**

la reflexión teórica sobre las buenas prácticas obstétricas, a la luz del pensamiento de la complejidad, da lugar a itinerarios formativos disruptivos, evolutivos y sostenibles, capaces de superar las barreras disciplinarias y establecer procesos horizontales, colegiados y sistémicos de intervención en obstetricia.

## INTRODUCTION

At first glance, some may wonder: what is the relationship between good obstetric practices and complexity thinking, or how this could be related to the Sustainable Development Goals? From the perspective of simplified thinking, which leads to the linearity of paths and processes, the possibilities of association are probably remote. However, the increasing public investments in research, public health policies, and care, more specifically with regard to good obstetric practices, are recognized. But what has really evolved in the field of obstetrics?

In Brazil, training in Obstetrics has been guided by public policies, investments in the training of specialist Obstetric Nurses, based on agreements signed between the Ministry of Health and universities, residencies in Obstetric Nursing, among other initiatives. In addition to enabling Nursing professionals, these initiatives were born with the purpose of reorganizing the work process and fostering a new obstetric culture, capable of expanding interactive possibilities, overcoming the segmentation of knowledge and transcending the interventionist obstetric model^(1,2)^.

However, how could we foster a new obstetric culture from the perspective of complexity thinking? Pregnancy, childbirth and birth constitute, par excellence, a complex phenomenon, due to the fact that they involve a complex network of interpersonal and interprofessional relationships and hierarchical conflicts during obstetric care. Under this approach, this complex and inseparable phenomenon “pregnancy, childbirth and birth” must be understood as a complex, dynamic, unpredictable adaptive system that interacts with itself and with the environment^(3)^.

Among the theoretical assumptions, complexity thinking comprises inseparability, complementarity and interconnectivity in interpersonal and interprofessional relationships. In an attempt to transcend reductionist Cartesian thinking, governed by inflexible hierarchy, separability and linearity, complexity thinking tends to rescue the existing relationships between different knowledges^(4)^ and enable flexible, expanded, multidimensional and systemic obstetric care.

Enabling a new obstetric culture, supported by complex adaptive systems, implies understanding the mother-child dyad in its uniqueness, complementarity, connectivity and inseparability; transcending the disciplinary barriers of the health area and achieving an integrative, collaborative and interprofessional thinking. It implies recognizing that paradigmatic transposition is associated with the ability to evolve in reflexivity, which includes contradictory and, apparently, antagonistic actions, to generate complex and systemic phenomena^(5,6)^.

The understanding of sustainable development leads to a path that considers current needs and is capable of promoting disruptive formative trajectories. This study aims to contribute to overcoming the technocratic, interventionist, and mechanistic model in obstetrics. It seeks interprofessionalism, which is based on the pillars of comprehensive care, patient safety, humanization of practices, and the promotion of the well-being of users and workers.

## OBJECTIVES

To reflect on good obstetric practices in light of complexity thinking.

## METHODS

This is a theoretical-reflective essay that considered classic bibliographic sources and contemporary authors who defend the evolutionary dynamics of complex adaptive systems and, in parallel, provide theoretical-systemic support to the complex and inseparable phenomenon of “pregnancy, childbirth, and birth”. This essay was conceived by researchers who are part of the Research and Studies Group in Social Entrepreneurship in Nursing - GEPESES, more specifically from provocations raised in the discipline “Construction of systemic thinking in maternal and child health”.

It was considered that the inseparability between the complex network of interpersonal and interprofessional relationships and hierarchical conflicts during obstetric care, in light of complexity thinking, confers a sense of integrality and complementarity, as well as greater safety and sustainability. The concept of complexity^(4,5)^, in its original conception, does not foresee a linear path of conception and analysis of care and management pathways in the field of obstetrics, but induces a prospective itinerary, in which the researcher must lead their own path, based on their context and lived experiences. Under this impulse, the proponents of this study initially focused, individually, on the search for and deepening of references that could broaden the theoretical perspective in relation to the object of investigation.

In a second phase, formative meetings were held to discuss the themes that served as axes for the refinement of the reflective themes. These meetings took place in a dynamic and participatory way, based on the creation of mind maps and infographics, in order to stimulate critical and prospective reflexivity. By synthesizing complex ideas into visual representations, the proponents were able to reframe their theoretical-practical experiences and validate the themes that make up the corpus of this study.

Conceived under the concept of complexity, this study conceives of “pregnancy, childbirth and birth” from a broader and systemic perspective, weaving together the experiences of learning, teaching, investigating, congregating and enabling a new look at the field of obstetrics. The induction of complexity thinking in the field of obstetrics is essential to a broader, contextualized, sustainable and interdependent understanding of the mother-child dyad^(7)^.

Because this study did not use primary sources, it was not reviewed by the Research Ethics Committee. However, scientific integrity and its ethical principles were considered.

## RESULTS

The theoretical reflection focuses on the following reflective themes: Complexity thinking as a guide for disruptive formative paths; and Complexity thinking as a guide for good evolutionary and sustainable obstetric practices.

### Complexity thinking as a guide of disruptive learning pathways

Innovation and the mobilization of disruptive processes, especially in initial and continuing education, require the transition from traditional models to critical-reflective learning ecosystems. Continuing to produce more of the same will not enable cultural advances, nor will it contribute to the necessary progress in health. Studies demonstrate that disruptive training, more precisely in continuing and permanent health education, constitutes a central element for questioning old paradigms and overcoming hegemonic interventionist approaches^(5)^.

Disruptive Continuing Education Programs are, therefore, capable of promoting greater engagement among Nursing/health professionals and inducing movements that awaken the necessary interconnectivity and complementarity of care practices. Continuing education in health, based on interactive and associative theoretical-methodological frameworks, such as complexity thinking, is capable of intuiting evolutionary, autonomous approaches committed to good sustainable practices in health^(8)^. Given the need to reconcile ideas and develop disruptive training pathways that stimulate critical-reflective thinking and enable complementary and interprofessional care processes^(8)^, it is essential to point out guiding paths. In this direction, reflective questions that induce new thinking among health professionals are presented in Chart 1. These questions emerged from the readings and discussions of the materials that supported the critical-reflective analysis of the proponents.

**Chart 1:** Reflective questions inducing new thinking among health professionals, Santa Maria, Rio Grande do Sul, Brazil, 2025

N	Training path	Reflective and evolutionary issues in obstetrics
1	Undergraduate level education	Is it possible to sensitize undergraduate professors to evidence-based training? Is it possible to establish horizontal and interdisciplinary approaches in initial teacher training? Is it possible to induce theoretical and practical experiences that foster student autonomy and leadership?
2	Specialized level training	Is it possible to establish horizontal and interdisciplinary approaches in training? Is it possible to encourage integrated practices among different health professionals? Is it possible to integrate theory and practice in professional settings?
3	On-the-job training	Is it possible to establish horizontal and interdisciplinary approaches in training? Is it possible to intuit dialogical paths and the exchange of experiences among health professionals? Is it possible to promote fruitful spaces for discussions of complex cases?
4	Lifelong learning	Is it possible to encourage the (re)construction of prospective knowledge and practices throughout life? Is it possible to leverage goals for participation in congresses, symposia, and other events? Is it possible to develop a career plan in obstetrics?

### Complexity thinking as a guide of good, evolving, and sustainable obstetric practices

Good obstetric practices demand an inclusive environment, human resources sensitive and flexible to change, horizontal intervention approaches, and person-centered care. As a flexible and dynamic adaptive system, good obstetric practices are not punctual, linear, and unilateral. Their dynamism and vitality occur to the extent that each human being is apprehended as a complex unit - singular and multidimensional. It also occurs to the extent that the different actors are recognized, valued, and considered in their core knowledge and from a network of complementary and inseparable attributions^(6)^.

**Figure 1:**
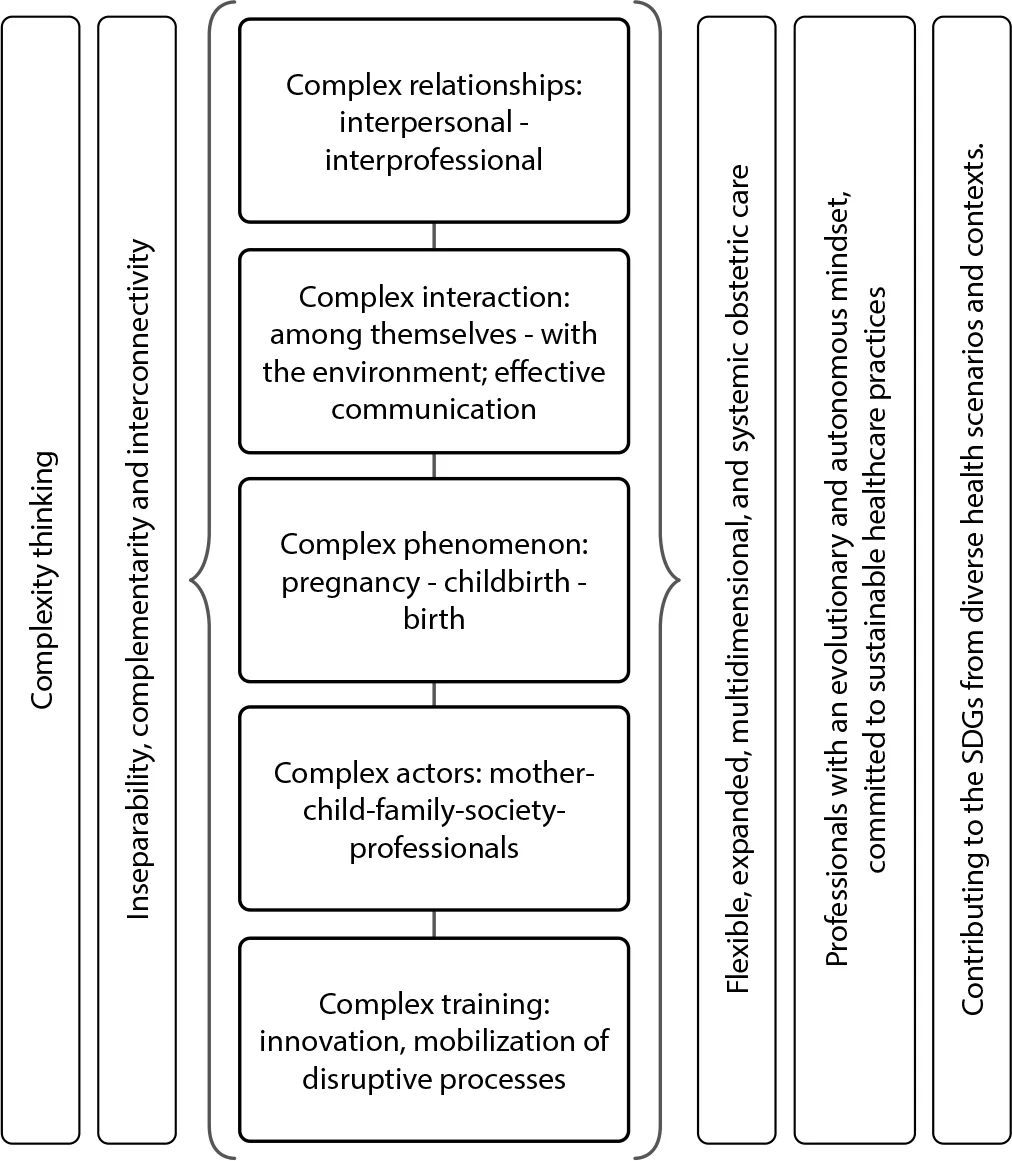
Good obstetric practices in light of complexity thinking, Santa Maria, Rio Grande do Sul, Brazil, 2025

As a guide for evolutionary and sustainable good obstetric practices, complexity thinking constitutes a prospective theoretical-strategic perspective in the context of training and work. Interventionist conduct governed by traditional Cartesian thinking has already shown that it is not possible to continue moving in this same direction. The promotion of good obstetric practices requires, based on Figure 1 below, professionals capable of reorganizing the disordered path and enabling a “new order”, as proposed by the author of complexity theory, capable of encompassing the uniqueness within the complexity of the whole, as well as the complex whole within the singularity of each person, professional, and system^(6,7)^.

By understanding pregnancy, childbirth, and birth as inseparable phenomena and integral parts of complex adaptive systems, it becomes possible to overcome disciplinary barriers, reduce hierarchical asymmetries, and promote horizontal, collegial care practices centered on the mother-child dyad.

## DISCUSSION

Complexity thinking must be grasped and understood as a fabric of heterogeneous elements inseparably associated, representing the paradoxical relationship between the one and the many, between the past, present and future^(5)^. This thinking is capable of associating and interconnecting seemingly dispersed, disordered and relegated knowledge, such as the humanitarian legacy of the forerunners of nursing.

Complexity thinking is, par excellence, a guide for disruptive formative paths, capable of enabling advances, transcending paradigms and establishing new movements towards the achievement of the Sustainable Development Goals. This thinking seeks to offer a new paradigm to obstetric practice that prospectively contemplates the dynamic and evolving nature of health care. It also recognizes the importance of interprofessional collaboration and effective communication in improving the quality of obstetric care^(5,6)^. The integration of the principles of complexity thinking into training pathways, both at the introductory level and in continuing health education processes, enables more flexible, interdependent, and complementary care approaches. This training dynamic promotes a qualified response to users and health systems, based on singular, multidimensional care that is consistent with real needs^(9)^.

Evolutionary-sustainable thinking, in light of complexity thinking, leads to life coherence in the personal, professional, and social spheres and suggests advances that transcend areas of knowledge, more specifically in the context of health. This way of thinking translates into an emancipatory movement that shifts from individual thinking to collective and collaborative action, in favor of greater quality, safety, and effectiveness, especially in the obstetric area^(4)^. Studies demonstrate that good obstetric practices, in light of the principles of complexity, broaden interprofessional perspectives, enable more horizontal relationships between professionals and users, and guarantee strategies that transcend interventionist practices, which are still strongly prevalent in Brazil. The same studies ensure that policies that induce best practices in obstetrics are not enough if professional conduct continues to reproduce specific and vertical styles and routines^(1,2)^.

Pregnancy, childbirth, and birth are, in themselves, complex phenomena and, therefore, understood in greater depth in light of complexity thinking. This process has led to the expansion of Nursing practices, especially in Primary Health Care worldwide. This reorganization of care has highlighted more horizontal relationships and interprofessional collaboration between Nurses, doctors, and other health professionals^(10)^.

Investing in Nursing professionals constitutes a promising strategy for the development of best practices in obstetrics. While medical professionals continue to be governed by the hegemonic framework of simplification - which leads to interventionist and often unprincipled obstetric practices - nurses are driven by the knowledge of complexity that, by excellence, leads to evolutionary paths and best obstetric practices^(2)^.

### Study limitations

In light of complexity thinking, knowledge is permanently unfinished and evolving. Similarly, complex adaptive systems are context-sensitive, which prevents the reflective propositions of this study from being applied as universal formulas. Thus, in line with the perspective of complexity - which rejects absolute neutrality - the scope of this study lies in its potential to promote disruptive educational pathways capable of inducing a new way of thinking about obstetric practices.

### Contributions to the field of nursing and health

The contributions of this study to the advancement of nursing science are related to the proposition of complex thinking in training pathways aimed at inducing good obstetric practices, in order to broaden professional perspectives and value the leading role of nursing in the development of horizontal and inclusive approaches. Another contribution is associated with fostering evolutionary thinking among nursing professionals, capable of contributing to the achievement of the Sustainable Development Goals.

## FINAL CONSIDERATIONS

The theoretical reflection carried out shows that complexity thinking constitutes a powerful source and guide for paradigmatic changes in obstetrics, and can favor disruptive, evolutionary and sustainable training paths.

This perspective expands the capacity of nursing professionals, as they lead structural transformations in care, aligning with the Sustainable Development Goals and the demands of safe, humanized and effective care. By breaking with interventionist and linear models, complexity thinking invites the construction of a new obstetric culture, based on interprofessionalism, respect for singularity and the integration of knowledge, generating positive impacts for health systems, for women, newborns and for society.

## Data Availability

The research data are available within the article.
