# Supplementary material for: Complexity thinking as a guide for good, evolving, and disruptive obstetric practices
Source: Rev Bras Enferm. 2026 Aug 21;79:e20250504. doi: 10.1590/0034-7167-2025-0504 (PMC13505562; doi:10.1590/0034-7167-2025-0504)
Supplement: Translation

# Translation: Pensamento da complexidade como indutor de boas práticas obstétricas evolutivas e disruptivas

**DOI:** 10.1590/0034-7167-2025-0504pt

**Authors:** Roseleia Regina Halmenschlager, Marli Terezinha Stein Backes, Janet Mercedes Arévalo-Ipanaqué, Dirce Stein Backes

**Affiliations:** I Universidade Franciscana. Santa Maria, Rio Grande do Sul, Brasil; II Universidade Federal de Santa Catarina. Florianópolis, Santa Catarina, Brasil; III Universidad Peruana Cayetano Heredia. Lima, Peru

**Keywords:** Dinâmica não Linear, Enfermagem Obstétrica, Equipe de Assistência ao Paciente, Identidade Profissional, Parto Normal.

## Abstract

**Objetivos:**

refletir sobre as boas práticas obstétricas à luz do pensamento da complexidade.

**Métodos:**

ensaio teórico-reflexivo que considerou fontes bibliográficas clássicas e de autores contemporâneos que defendem a dinâmica evolutiva dos sistemas adaptativos e, paralelamente, conferem sustentação teórico-sistêmica ao fenômeno complexo e indissociável “gestação, parto e nascimento”.

**Resultados:**

ao compreender a gestação, o parto e o nascimento como fenômenos indissociáveis e integrantes de sistemas adaptativos complexos, torna-se possível transpor barreiras disciplinares, reduzir assimetrias hierárquicas e promover práticas assistenciais horizontalizadas, colegiadas e centradas na díade mãe-filho.

**Considerações Finais:**

a reflexão teórica acerca das boas práticas obstétricas, à luz do pensamento da complexidade, suscita percursos formativos disruptivos, evolutivos e sustentáveis, capazes de transpor as barreiras disciplinares e estabelecer processos horizontalizados, colegiados e sistêmicos de intervenção em obstetrícia.

## INTRODUÇÃO

À primeira vista há quem se pergunte: qual a relação das boas práticas obstétricas com o pensamento da complexidade ou qual a sua relação com os Objetivos de Desenvolvimento Sustentável? Sob a ótica do pensamento simplificado, que conduz à linearidade dos percursos e processos, as possibilidades de associação são, provavelmente, remotas. Reconhece-se, no entanto, os crescentes investimentos públicos na pesquisa, nas políticas públicas de saúde e na assistência, sobretudo no que se refere às boas práticas obstétricas. Mas o que realmente evoluiu no campo da obstetrícia?

Em âmbito de Brasil, a formação em Obstetrícia tem sido induzida por meio de políticas públicas, investimentos na formação de Enfermeiras Obstétricas especialistas, a partir de convênios firmados entre o Minstério da Saúde com universidades, residências em Enfermagem Obstétrica, dentre outras iniciativas. Além de habilitar profissionais Enfermeiros, essas iniciativas nasceram com o propósito de reordenar o processo de trabalho e suscitar nova cultura obstétrica, capaz de espandir as possibilidades interativas, superar a segmentação do conhecimento e transcender o modelo obstétrico intervencionista^(1,2)^.

Como suscitar, todavia, uma nova cultura obstétrica na perspectiva do pensamento da complexidade? A gestação, parto e nascimento constituem-se, por excelência, em fenômeno complexo, pelo fato de envolverem uma complexa rede de relacionamentos interpessoais, interprofissionais e conflitos hierárquicos na condução dos cuidados obstétricos. Sob esse enfoque, esse fenômeno complexo e indissociável “gestação, parto e nascimento” deve ser apreendido como sistema adaptativo complexo, dinâmico, imprevisível, que interage entre si e com o ambiente^(3)^.

Dentre os pressupostos teóricos, o pensamento da complexidade concebe a indissociabilidade, a complementariedade e a interconectividade nas relações interpessoais e interprofissionais. Na tentativa de transcender o pensamento cartesiano reducionista, regido pela hierarquia inflexível, pela separabilidade e linearidade, o pensamento da complexidade tende a resgatar as relações existentes entre os diferentes saberes^(4)^ e possibilitar uma assistência obstétrica flexível, ampliada, multidimensional e sistêmica.

Possibilitar uma nova cultura obstétrica, sustentada em sistemas adaptativos complexos, implica em apreender a díade mãe-filho em sua unicidade, complementariedade, conectividade e indissociabilidade; em transcender as barreiras disciplinares da área da saúde e alcançar um pensamento integrador, colaborativo e interprofissional. Implica em reconhecer que a transposição paradigmática está associada à capacidade de evoluir na reflexibilidade, que comporta ações contraditórias e, aparentemente, antagônicas, para gerar fenômenos complexos e sistêmicos^(5,6)^.

A compreensão de desenvolvimento sustentável induz a um percurso que considera as necessidades atuais e é capaz de promover trajetórias formativas disruptivas. Este estudo propõe-se a contribuir para a superação do modelo tecnocrático, intervencionista e mecanicista em obstetrícia. Busca-se a interprofissionalidade, que tem como pilares a integralidade da atenção, a segurança do paciente, a humanização das práticas e a promoção do bem-estar de usuários e trabalhadores.

## OBJETIVOS

Refletir sobre as boas práticas obstétricas à luz do pensamento da complexidade.

## MÉTODOS

Trata-se de um ensaio teórico-reflexivo que considerou fontes bibliográficas clássicas e de autores contemporâneos que defendem a dinâmica evolutiva dos sistemas adaptativos complexos e, paralelamente, conferem sustentação teórico-sistêmica ao fenômeno complexo e indissociável “gestação, parto e nascimento”. O presente ensaio foi idealizado por pesquisadores que integram o Grupo de Pesquisa e Estudos em Empreendedorismo Social da Enfermagem - GEPESES, mais especificamente a partir de provocações suscitadas na disciplina “Construção do pensamento sistêmico em saúde materno-infantil”. Considerou-se que a indissociabilidade entre a complexa rede de relacionamentos interpessoais, interprofissionais e conflitos hierárquicos na condução dos cuidados obstétricos, à luz do pensamento da complexidade, confere sentido de integralidade e complementariedade, além de maior segurança e sustentabilidade.

O pensamento da complexidade^(4,5)^, em sua concepção originária, não prevê um percurso linear de concepção e análise dos percursos assistenciais e de gestão no campo da obstetrícia, mas induz a um itinerário prospectivo, no qual o investigador deve protagonizar o seu próprio caminho, a partir de seu contexto e experiências do vivido. Sob esse impulso, os proponentes deste estudo debruçaram-se, inicialmente, de modo individual, na busca e no aprofundamento de referenciais que pudessem ampliar a perspectiva teórica em relação ao objeto de investigação.

Realizou-se, num segundo momento, encontros formativos para discutir os temas que serviram de eixos para o refinamento das temáticas reflexivas. Esses encontros ocorreram de forma dinâmica e participativa, a partir da criação de mapas mentais e infográficos, de modo a estimular a reflexividade crítica e prospectiva. Ao sintetizar ideias complexas em representações visuais, os proponentes puderam ressignificar suas experiências teórico-práticas e validar as temáticas que compõem o *corpus* deste estudo.

Idealizado sob o pensamento da complexidade, o presente estudo concebe a “gestação, parto e nascimento” em perspectiva ampliada e sistêmica, ao tecer em conjunto as experiências do vivido no aprender, ensinar, investigar, congregar e possibilitar um novo olhar sobre o campo da obstetrícia. A indução do pensamento da complexidade no campo da obstetrícia é essencial à compreensão ampliada, contextualizada, sustentável e interdependente da díade mãe-filho^(7)^.

Pelo fato de não utilizar fontes primárias, este estudo não passou pelo Comitê de Ética em Pesquisa. Considerou-se, contudo, os princípios éticos da integridade científica.

## RESULTADOS

A reflexão teórica centra-se nas seguintes temáticas reflexivas: Pensamento da complexidade como indutor de percursos formativos disruptivos; e Pensamento da complexidade como propulsor de boas práticas obstétricas evolutivas e sustentáveis.

### Pensamento da complexidade como indutor de percursos formativos disruptivos

A inovação e a mobilização de processos disruptivos, especialmente, na formação inicial e continuada requer a transição de modelos tradicionais para ecossistemas de aprendizagem crítico-reflexiva. Seguir produzindo mais do mesmo não possibilitará avanços culturais e nem mesmo contribuirá para os necessários progressos na saúde. Estudos demonstram que a formação disruptiva, mais precisamente na formação continuada e permanente em saúde, constitui-se como elemento central para questionar velhos paradigmas e superar abordagens intervencionistas hegemônicas^(5)^.

Programas de Educação Permanente disruptivos são, portanto, capazes de promover maior engajamento entre os profissionais de Enfermagem/saúde e induzir movimentos que despertem à necessária interconectividade e complementariedade das práticas assistenciais. A educação permanente em saúde, com base em referenciais teórico-metodológicos interativos e associativos, a exemplo do pensamento da complexidade, são capazes de intuir abordagens evolutivas, autônomas e comprometidas com as boas práticas sustentáveis em saúde^(8)^.

Face à necessidade de conciliar ideias e desenvolver percursos formativos disruptivos que estimulem a reflexão crítico-reflexiva e possibilitem processos assistenciais complementares e interprofissionais^(8)^ é fundamental que se aponte caminhos orientadores. Nessa direção, apresenta-se, no Quadro 1, questões reflexivas indutoras de um novo pensamento entre os profissionais de saúde. Essas questões emergiram das leituras e discussões dos materiais que subsidiaram a análise crítico-reflexiva dos proponentes.

**Quadro 1:** Questões reflexivas indutoras de um novo pensamento entre os profissionais de saúde, Santa Maria, Rio Grande do Sul, Brasil, 2025

N	Percurso formativo	Questões reflexivas e evolutivas em obstetrícia
1	Formação em nível de graduação	É possível sensibilizar os professores da graduação para uma formação baseada em evidências científicas? É possível estabelecer abordagens horizontalizadas e interdisciplinares na formação inicial? É possível induzir vivências teórico-práticas que despertem a autonomia e o protagonismo do estudante?
2	Formação em nível de especialização	É possível estabelecer abordagens horizontalizadas e interdisciplinares na formação? É possível induzir práticas integradas entre os diferentes profissionais da saúde? É possível integrar teoria à prática nos cenários de atuação profissional?
3	Formação em serviço	É possível estabelecer abordagens horizontalizadas e interdisciplinares na formação? É possível intuir percursos dialógicos e a troca de experiências entre os profissionais de saúde? É possível promover espaços profícuos de discussões de casos complexos?
4	Formação ao longo da vida	É possível estimular à (re)construção de saberes e práticas prospectivas ao longo da vida? É possível alavancar metas de participação em congressos, simpósios e outros? É possível desenvolver um plano de carreira em obstetrícia?

### Pensamento da complexidade como propulsor de boas práticas obstétricas evolutivas e sustentáveis

As boas práticas obstétricas demandam ambiência agregadora, recursos humanos sensíveis e flexíveis às mudanças, abordagens de intervenção horizontalizadas e um cuidado centrado na pessoa. Enquanto sistema adaptativo flexível e dinâmico, as boas práticas obstétricas não são pontuais, lineares e unilaterais. A sua dinâmica e vitalidade ocorre na medida em que cada ser humano é apreendido como unidade complexa - singular e multidimensional. Ocorre, ainda, na medida em que os diferentes atores são reconhecidos, valorizados e considerados em seus saberes nucleadores e a partir de uma rede de atribuições complementares e indissociáveis^(6)^.

Como propulsor de boas práticas obstétricas evolutivas e sustentáveis, o pensamento da complexidade se constitui em perspectiva teórico-estratégica prospectiva em âmbito da formação e do trabalho. As condutas intervencionistas e regidas pelo pensamento tradicional cartesiano já deram mostras de que não é possível seguir caminhando nessa mesma direção. A indução de boas práticas obstétricas requer, com base na Figura 1, a seguir, profissionais capazes de reorganizar o percurso desordenado e possibilitar uma “nova ordem”, conforme proposto pelo autor do pensamento da complexidade, capaz de abarcar a unicidade na complexidade do todo, assim como o todo complexo na singularidade de cada pessoa, profissional, sistema^(6,7)^.

**Figura 1:** Boas práticas obstétricas à luz do pensamento da complexidade, no intuito de contribuir para o alcance dos Objetivos de Desenvolvimento Sustentável

Ao compreender a gestação, o parto e o nascimento como fenômenos indissociáveis e integrantes de sistemas adaptativos complexos, torna-se possível transpor barreiras disciplinares, reduzir assimetrias hierárquicas e promover práticas assistenciais horizontalizadas, colegiadas e centradas na díade mãe-filho.

## DISCUSSÃO

O pensamento da complexidade deve ser apreendido e compreendido como um tecido de elementos heterogêneos inseparavelmente associados, que representam a relação paradoxal entre o uno e o múltiplo, entre o passado, presente e futuro^(5)^. Esse pensamento é capaz de associar e interligar saberes aparentemente dispersos, desordenados e relegados a um segundo plano, tais como o legado humanitário das precursoras de enfermagem.

O pensamento da complexidade é, por excelência, indutor de percursos formativos disruptivos, capazes de possibilitar avanços, transcender paradigmas e instaurar novos movimentos em direção ao alcance dos Objetivos de Desenvolvimento Sustentável. Esse pensamento busca oferecer um novo paradigma à prática obstétrica que contemple, prospectivamente, a natureza dinâmica e evolutiva da assistência à saúde. Reconhece, igualmente, a importância da colaboração interprofissional e da comunicação eficaz à melhoria da qualidade da assistência obstétrica^(5,6)^.

A integração dos princípios do pensamento da complexidade aos percursos formativos, tanto em âmbito de iniciação quanto em processos de educação permanente em saúde, viabiliza abordagens assistenciais mais flexíveis, interdependentes e complementares. Tal dinâmica formativa promove uma resposta qualificada aos usuários e aos sistemas de saúde, pautada em um cuidado singular, multidimensional e condizente com as reais necessidades^(9)^.

O pensamento evolutivo-sustentável, à luz do pensamento da complexidade, induz à coerência de vida em âmbito pessoal, profissional, social e sugestiona avanços que transcendem áreas de conhecimento, mais especificamente no contexto da saúde. Essa forma de pensar traduz-se em um movimento emancipatório que se desloca do pensar individual ao agir coletivo e colaborativo, em prol de mais qualidade, segurança e efetividade, especialmente na área obstétrica^(4)^.

Estudos demonstram que as boas práticas obstétricas, à luz dos preceitos da complexidade, ampliam perspectivas interprofissionais, possibilitam relações mais horizontalizadas entre profissionais e usuários e garantem estratégias que transcendem as práticas intervencionistas, ainda fortemente vigentes no Brasil. Os mesmos estudos asseguram que não bastam políticas indutoras de melhores práticas em obstetrícia se as condutas profissionais continuam a reproduzir estilos e rotinas pontuais e verticalizadas^(1,2)^.

A gestação, o parto e o nascimento são, por si só, fenômenos complexos e, portanto, compreendidos em maior profundidade à luz do pensamento da complexidade. Esse processo conduziu à expansão das práticas de Enfermagem, especialmente na Atenção Primária à Saúde em todo o mundo. Tal reorganização do cuidado tem evidenciado relações mais horizontais e a colaboração interprofissional entre Enfermeiros, médicos e demais profissionais de saúde^(10)^.

Investir nos profissionais de Enfermagem constitui-se em uma estratégia promissora para o desenvolvimento de melhores práticas em obstetrícia. Enquanto os profissionais médicos seguem regidos pelo referencial hegemônico da simplificação - que induz a práticas obstétricas intervencionistas e, muitas vezes, sem critérios - os enfermeiros movem-se pelo saber da complexidade que, por excelência, conduz a percursos evolutivos e às melhores práticas obstétricas^(2)^.

### Limitações do estudo

À luz do pensamento da complexidade, o conhecimento é permanentemente inacabado e evolutivo. Da mesma forma, sistemas adaptativos complexos são sensíveis ao contexto, o que impede que as proposições reflexivas deste estudo sejam aplicadas como fórmulas universais. Assim, em consonância com a perspectiva da complexidade - que rejeita a neutralidade absoluta - o alcance deste estudo reside na sua potencialidade de promover percursos formativos disruptivos, capazes de induzir um novo pensar sobre as práticas obstétricas.

### Contribuições para a área de enfermagem e saúde

As contribuições deste estudo para o avanço da ciência de Enfermagem estão relacionadas à proposição do pensamento complexo em percursos formativos de indução das boas práticas obstétricas, de modo a ampliar as perspectivas profissionais e valorizar o protagonismo da Enfermagem no desenvolvimento de abordagens horizontalizadas e inclusivas. Outra contribuição está associada ao fomento de um pensamento evolutivo entre os profissionais de Enfermagem, capaz de contribuir para o alcance dos Objetivos de Desenvolvimento Sustentável.

## CONSIDERAÇÕES FINAIS

A reflexão teórica realizada evidencia que o pensamento da complexidade se constitui em potente indutor e propulsor de mudanças paradigmáticas na obstetrícia, podendo favorecer percursos formativos disruptivos, evolutivos e sustentáveis.

Essa perspectiva amplia a capacidade dos profissionais de Enfermagem, ao protagonizarem transformações estruturais no cuidado, alinhando-se aos Objetivos de Desenvolvimento Sustentável e às demandas de uma assistência segura, humanizada e efetiva. Ao romper com modelos intervencionistas e lineares, o pensamento da complexidade convida à construção de uma nova cultura obstétrica, pautada na interprofissionalidade, no respeito à singularidade e na integração de saberes, gerando impactos positivos para os sistemas de saúde, para as mulheres, neonatos e para a sociedade.

## Data Availability

Os dados de pesquisa estão disponíveis no corpo do artigo.
